# Safety Evaluation of a Prototypical Diazirine-Based Covalent Crosslinker and Molecular Adhesive

**DOI:** 10.1177/10915818231215692

**Published:** 2023-11-21

**Authors:** Miranda J. Baran, Rebecca Hof, Angelique Groot, Irene Eurlings, Jet Gijsbrechts, Britt de Jong, Jeremy E. Wulff

**Affiliations:** 1 XLYNX Materials Inc., Victoria, BC, Canada; 2 Department of Chemistry, 8205 University of Victoria, Victoria, BC, Canada; 3 Centre for Advanced Materials and Related Technology (CAMTEC), 8205 University of Victoria, Victoria, BC, Canada; 4 26135 Charles River Laboratories Den Bosch BV, ‘s-Hertogenbosch, The Netherlands

**Keywords:** diazirines, polymer crosslinkers, adhesives, toxicity studies

## Abstract

*bis*-Diazirine reagents are increasingly being used as polymer crosslinkers, adhesives, and photopatterning agents in the materials sciences literature, but little effort has been made thus far to document their chemical safety profile. Here, we describe the results of a detailed toxicity assessment of a representative *bis*-diazirine. Safety was evaluated by a series of in vitro assays, which found the product to be non-mutagenic in bacterial tester strains TA98 and TA100, non-corrosive and non-irritating to skin, and requiring no classification for eye irritation or serious damage. While in vitro tests do not capture the integrated whole animal system, and thus cannot completely rule out the possibility of adverse responses, the results of this study suggest a desirable safety profile for *bis*-diazirine reagents and provide a solid foundation upon which to add in vivo assessment of safety risk and dose-response studies.

## Introduction

Polypropylene (PP) and polyethylene (PE) together constitute the most abundantly manufactured non-fibre plastics worldwide, owing to their good mechanical strength, low density, and low cost.^1^ Despite being widely applied in fields ranging from automotive manufacturing, to packaging, to surgical implants, these plastics are poorly suited to bonding with adhesives as a consequence of their low surface energy.^2–5^ Conventional adhesives such as glues and epoxies rely upon hydrogen bonding, van der Waals interactions, and polymer entanglement to create adhesion.^6^ Since PP and PE are comprised of non-polar carbon–carbon and carbon–hydrogen bonds, they do not participate in hydrogen bonding, resulting in adhesion failure unless costly high-energy treatments (eg corona discharge) are applied to prime the surface.^7,8^

**Figure 1.**
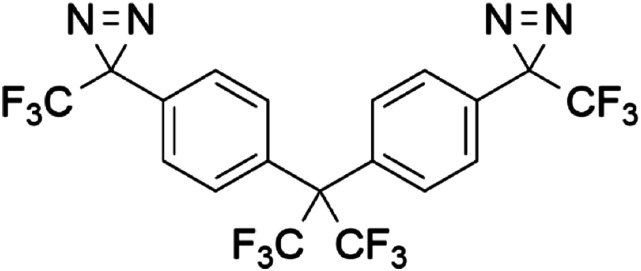
Structure of BondLynx^©^ (CAS: 2409741-22-4, IUPAC name: 2,2-*bis-*[4-[3-(trifluoromethyl)-3*H*-diazirin-3-yl]phenyl]hexafluoropropane).

In 2019, the perfluoropropyl-bridged *bis*-(trifluoromethyl phenyldiazirine) BondLynx^©^ (Figure 1) was described as a near-universal crosslinker for aliphatic polymers.^9^ The key to the performance of BondLynx is the reactivity of the two diazirine motifs: strained three-membered rings that incorporate two doubly bound nitrogen atoms.^10^ Under modest heat (80-110°C) or UV light (365-405 nm), diazirine groups can expel a molecule of nitrogen gas to reveal a reactive carbene species, which is then capable of undergoing rapid C–H, O–H, or N–H insertion with nearby polymer chains, forming strong covalent bonds.^11^ When two diazirine groups are linked together, as in the case of BondLynx, new bonds can be formed between polymer chains. This crosslinking process can result in improved mechanical properties as well as increased high-temperature performance. At the same time, simply painting *bis*-diazirine BondLynx between two pieces of polyolefin materials, and then triggering diazirine activation by heating, results in the formation of new bonds across the polymer–polymer interface. The result is strong, molecular level adhesion. The non-specific nature of the carbene insertion means that adhesion is equally successful for low- or high-surface energy polymers, so long as aliphatic C–H bonds are available for reaction.

Following the initial disclosure of BondLynx, several structurally related *bis*- (and occasionally *tetrakis*-) trifluoromethyl phenyldiazirines have been described in the materials sciences literature.^12–15^ These reagents have been used for a wide array of applications, including adhesion of low-surface energy plastics and elastomers,^13,16^ photopatterning of lipophilic polymers for organic electronics applications,^17–21^ direct photoprinting of electroluminescent quantum dot aggregates,^22^ and upgrading the mechanical performance of both fabrics^14,23^ and coatings.^24^ Relatively small amounts of material are required to produce significant changes in mechanical properties, for example more than doubling the tear resistance of UHMWPE fabric at 1 wt% loading.^9^ BondLynx is a crystalline solid with a melting point of 34°C, allowing it to be handled as a solid or liquid with relative ease, and is typically dissolved in a low boiling point solvent (eg pentane or diethyl ether) to produce a thin, uniform layer.

While considerable effort has been directed toward establishing the thermal stability of novel *bis*-diazirine reagents (ensuring that they are not shock sensitive or prone to explosion),^9,13,14^ thus far there have been no studies investigating their toxicity. While the intended uses of BondLynx as a crosslinker, adhesive, or adhesive primer do not call for direct human exposure, the risk of incidental exposure to the skin and eyes during application indicate a need to understand potential hazards to the user. Accordingly, in this report, we assess the toxicity of the prototypical *bis*-diazirine (2,2-*bis-*[4-[3-(trifluoromethyl)-3*H*-diazirin-3-yl]phenyl]hexafluoropropane, BondLynx) through a series of in vitro assays for mutagenesis, phototoxicity, skin corrosion, skin irritation, and eye hazard studies. Chemical safety assessment by in vitro assay requires the use of cells or organoids that closely mimic the physiological environment of in vivo systems.^25^ The battery of tests performed herein all fall under the *Guidelines for the Testing of Chemicals* outlined by the Organisation for Economic Co-operation and Development (OECD): Test No. 471, 432, 439, 431, and 437, respectively. These tests, which have been extensively validated, offer an efficient means of identifying potential hazards for further investigation. Nevertheless, they cannot fully capture the whole-animal system, and should therefore be complemented by in vivo safety assessment in the future.

BondLynx was determined to be non-mutagenic by the *Salmonella typhimurium* reverse mutation assay in bacterial tester strains TA98 and TA100, non-phototoxic by in vitro 3T3 NRU phototoxicity test with no cytotoxicity observed in either the presence or absence of UV light, non-corrosive and non-irritating to skin by in vitro skin corrosion and irritation tests and requires no classification for eye irritation or serious eye damage based on the Bovine Corneal Opacity and Permeability test (BCOP test).

## Materials and Methods

### Test Substance Production and Analysis

BondLynx was prepared according to reported procedures from 4,4’-(perfluoropropane-2,2-diyl)dibenzoic acid.^9^ The structure and purity were confirmed to match literature standards by ^1^H-NMR, ^19^F-NMR, and UPLC (Supplementary Figures S1–S3).

All biological experiments were performed under contract by Charles River Laboratories Den Bosch BV.

### Bacterial Reverse Mutation Test

The reverse mutation test was performed to determine the potential of BondLynx and/or its metabolites to induce reverse mutations at the histidine locus of *Salmonella typhimurium*.^26,27^ The test employs strains of *Salmonella typhimurium* carrying mutations in the genes involved in histidine production, such that they are only able to grow in histidine supplemented culture media. Upon exposure to mutagenic materials, these mutations may be reversed, thereby restoring the ability to grow in histidine-free media. When grown in the presence of the test compound on media with a small amount of histidine, tester strains grow briefly to allow the opportunity for mutation, followed by proliferation of revertant colonies upon depletion of the provided histidine. Thus, the number of revertant colonies following incubation is indicative of the ability of the test compound to induce mutations.

Tests were performed on two strains of the bacteria (TA98 and TA100) in the absence and presence of an exogenous mammalian metabolic activation system (5% v/v S9). Positive controls and BondLynx were suspended in dimethyl sulfoxide (DMSO) for testing, and DMSO served as the vehicle control. Positive controls consisted of 2-nitrofluorene (10 μg/plate) for the TA98 strain without S9, methyl mesylate (650 μg/plate) for the TA100 strain without S9, and 2-aminoanthracene for both strains with S9 (1 μg/plate for TA98 and 5 μg/plate for TA100). Eight concentrations of BondLynx (1.7, 5.4, 17, 52, 164, 512, 1600 and 5000 μg/plate) were tested in triplicate. The vehicle control was tested in triplicate and the positive controls were tested singular.

Test solutions were prepared by pre-incubating .1 mL fresh bacterial culture (10^9^ cells/mL), .1 mL of a BondLynx dilution or control solution, and .5 mL of S9 mix or .1 M phosphate buffer for 30 min before adding to 3 mL of molten (45 ± 2°C) top agar. After mixing in a vortex, agar solutions were poured onto selective agar plates. Upon solidification, the prepared plates were inverted and incubated at 37.0 ± 1.0°C for 48 ± 4 h. Following incubation, revertant colonies were counted with a Sorcerer Colony Counter. The condition of the bacterial background lawn was evaluated macroscopically and/or microscopically using a dissecting microscope when considered necessary. Precipitate of BondLynx, while observed at some higher concentrations, was minimal and did not interfere with automated counting.

### Cytotoxicity and Phototoxicity in 3T3 Fibroblasts Using the Neutral Red Uptake Assay

The 3T3 Neutral Red uptake assay was used to evaluate the phototoxicity of BondLynx. The NRU assay makes use of Neutral Red dye, a weak cationic dye that penetrates the cell membrane by non-ionic passive diffusion and accumulates in lysosomes. Cytotoxicity of the test material is evaluated by measuring the OD_540_ to assess the uptake of Neutral Red dye.^28,29^ Only viable cells uptake Neutral Red dye as a result of pH gradients in the lysosomes, and thus more dye uptake, observed as a higher OD_540_, is indicative of more living cells, and cell viability following exposure to a test compound can be taken as the OD_540_ of treated relative to untreated cells. By measuring cytotoxicity in the absence vs presence of UV and visible light, the phototoxicity of the test material can be determined.^30,31^

Tests were performed using Balb/c 3T3 fibroblasts (clone 31) which were cultured in Dulbecco’s Modified Eagle Medium (DMEM) supplemented with heat-inactivated (56°C; 30 min) newborn foetal calf serum (20% v/v), L-glutamine (2 mM), penicillin (50 u/mL), and streptomycin (50 μg/mL). Incubations were performed in the dark at 36.8-37.4°C, in an atmosphere with 60%-93% humidity containing 5.0 ± .5% CO_2_ in air, unless otherwise stated. Cells were routinely trypsinized and passaged, at least once weekly, with a split ratio 1:3 to 1:4 to achieve a near confluent culture.

The positive control was anthracene in DMSO; dilutions were prepared from a 3.16 mg/mL stock for testing concentrations of .0100, .0316, .100, .316, 1.00, 3.16, 10.0, and 31.6 μg/mL. The negative control was sodium dodecyl sulphate in EBSS (Earle’s Balanced Salt Solution) medium; dilutions were prepared from a 3.16 mg/mL stock for testing concentrations of .100, .316, 1.00, 3.16, 10.0, 31.6, 100, and 316 μg/mL. Stock solutions of both controls were freshly prepared on the day of testing. The vehicle control was 1% DMSO in EBSS for the positive control, EBSS for the negative control, and .5% ethanol in EBSS for BondLynx. BondLynx was dissolved in ethanol for testing. Eight concentrations of BondLynx were prepared from a fresh (<2 h old) 200 mg/mL stock for testing concentrations of 1000, 316, 100, 31.6, 10.0, 3.16, 1.00, and .316 μg/mL. All controls and BondLynx were handled in the dark and tested in 8-fold.

In preparation for testing, cultures of 3T3 fibroblasts were trypsinized and resuspended in culture medium. Aliquots of 100 μL were seeded in the first ten columns of a 96-well plate, at a density of 15,000 cells/well, and incubated for 40-48 h to achieve 46%-60% confluency. A total of six plates were prepared in this manner – two each for the positive control, the negative control, and BondLynx. Following incubation, the cell culture medium was removed, and the wells washed with DPBS (Dulbecco’s phosphate-buffered saline). A 200 μL aliquot of each dilution of the test compound (BondLynx or a control) was added the first eight columns, and the appropriate vehicle control was added to the last four columns. All plates were incubated an additional 60 min before irradiating one plate for each test compound (+Irr) in a solar simulator with a WG320 nm filter for 22 min (equivalent to 5 J/cm^2^ UV-A), while the second plate for each test compound (–Irr) was kept in the dark at room temperature. Subsequently, the test solutions were removed from the plates, and the cells were washed once with DPBS and suspended in 200 μL of supplemented DMEM to incubate for an additional 20-24 h.

Immediately following incubation, the confluency of each plate was visually assessed, and the culture medium was replaced with 100 μL of Neutral Red dye (50 μg/mL). The plates were incubated another 3.5 h before removing the dye, washing with 150 μL of DPBS, and shaking for 20-40 min in 150 μL Neutral Red destain solution (ethanol:acetic acid:distilled water, 50:1:49). The optical density at 540 nm was measured for each well and corrected by subtracting the appropriate blank.

Where possible, the concentration of the test compound resulting in 50% inhibition of the Neutral Red uptake (IC_50_ value) in the presence (+Irr) and absence (–Irr) of UV-A irradiation was determined by plotting the logarithm of the test item concentration vs the extent of inhibition. Plots were fitted with a sigmoidal curve using Prism version 4.03 (GraphPad Software, San Diego, USA) according to the equation:

$${Corrected\;OD}_{540}=\frac{100}{1+10^{(\left(\log\left({IC}_{50}\right)-\;\log\left(concentration\right)\times HillSlope\right)}}$$

Where the IC_50_ could be measured, the Photo Irritation Factor (PIF) was calculated by dividing the non-irradiated IC_50_ by the irradiated IC_50_.

### Skin Corrosion Test Using a Human Skin Model

The skin corrosion test was performed to assess the cytotoxic effects of BondLynx immediately following short-term exposure to the stratum corneum of the epidermis using an in vitro skin model.^32–34^ Cytotoxicity to the skin model was expressed as the reduction of mitochondrial dehydrogenase activity measured by formazan production from 3-(4,5-dimethylthiazol-2-yl)-2,5-diphenyl tetrazolium bromide (MTT). To ensure that the presence of BondLynx did not interfere with the test system, it was checked for direct reduction of MTT and colour interference. BondLynx was checked for direct MTT reduction by incubating at least 50 mg of BondLynx with 1 mL MTT for 3 h to ensure that no blue/purple colour change or precipitate occurred. BondLynx was tested for colour interference by incubating at least 50 mg of BondLynx in water for 1 h and isopropanol for 2-3 h, centrifuging for 30 s at 16,000 g, and measuring the OD_570_ to ensure an optical density ≤.08 (after subtracting a Milli-Q water blank).

Tests were performed using the EpiDerm Skin Model, which consists of normal, human-derived epidermal keratinocytes cultured to on polycarbonate membranes in 10 mm cell culture inserts to form a multilayered, highly differentiated model of the human epidermis arranged in patterns analogous to those found in vivo.^35,36^ Incubations were performed at 37.0-37.3°C, in an atmosphere with 77%-95% humidity containing 5.0 ± .5% CO_2_ in air unless otherwise stated. The positive control was 8.0 N aqueous potassium hydroxide (KOH) solution, and the negative control was Milli-Q water. BondLynx was applied directly to the test system as a solid and spread to match the size of the tissue. Tests were performed in duplicate.

Skin tissue samples were refrigerated for 1 day upon receipt before placing in a six-well plate with .9 mL supplemented DMEM (just enough to reach the bottom of the tissue) 1 h before testing. A total of four samples were prepared per test item by applying 50 μL of the positive or negative control, or 25 μL of Milli-Q water (to moisten the tissue and ensure close contact) followed by 34.2-39.8 mg of BondLynx. Two samples of each test item were incubated for 3 min at room temperature, while the remaining samples were incubated for 1 h. After exposure, tissues were washed with phosphate buffered saline, dried carefully, and stored in 300 μL DMEM in a 24 well plate until the full six-well plate was dosed and rinsed.

Cell viability after incubation was determined by replacing the DMEM with 300 μL MTT in supplemented DMEM (1 mg/mL) and incubating for 3 h. Tissues were then washed with phosphate buffer saline, and formazan was extracted with 2 mL isopropanol overnight. The amount of extracted formazan was measured in triplicate for each sample by the OD_570_.

Optical density readings were corrected by subtracting the mean blank OD_570_. Cell viability was calculated as the percentage of the mean corrected OD_570_ for the test compound relative to the mean OD_570_ for the negative control (% Viability = OD_test_/OD_negative control_ × 100%).

### Skin Irritation Test Using a Human Skin Model

The skin irritation test was performed to assess the delayed cytotoxic effects of BondLynx following short-term exposure to the stratum corneum of the epidermis using an in vitro skin model.^37,38^ Cytotoxicity to the skin model was expressed as the reduction of mitochondrial dehydrogenase activity measured by formazan production from MTT.^39^ To ensure that the presence of BondLynx did not interfere with the test system, direct MTT reduction and colour interference were ruled out as was shown in the skin corrosion test.

Tests were performed using the EPISKIN Small Model^TM^ (.38 cm^2^), a three-dimensional human epidermis model consisting of adult human derived epidermal keratinocytes which were seeded onto a dermal substitute consisting of a collagen type I matrix coated with type IV collagen resulting in a highly differentiated and stratified epidermis model.^35,40–44^ Incubations were performed at 36.3-37.3°C, in an atmosphere with 81%-95% humidity containing 5.0 ± .5% CO_2_ in air unless otherwise stated. The positive control was 5% sodium dodecyl sulphate in phosphate buffered saline and the negative control was phosphate buffered saline. BondLynx was applied directly to the test system as a solid and spread to match the size of the tissue. Tests were performed in triplicate.

Skin tissue samples were transferred to 12-well plates upon receipt and pre-incubated with pre-warmed Maintenance Medium (Skinethic Laboratories) for approximately 23 hours. A total of three samples were prepared for each test item by moistening the skin with 5 μL Milli-Q water and then applying 25 μL of the positive or negative control, or 11.2-16.2 mg of BondLynx. Samples were incubated for 15 ± .5 min at room temperature, and the positive control was re-spread halfway through the contact period. Following exposure, samples were rinsed with phosphate buffered saline until no test item remain, dried carefully, and placed in a new 2 mL well with pre-warmed Maintenance Medium to incubate for 42 h.

After incubation, cell culture inserts were dried carefully, transferred into a 12-well plate prefilled with 2 mL MTT in phosphate buffered saline (.3 mg/mL), and incubated for 3 h. Tissue samples were then dried, and a biopsy punch was taken. The epidermis was separated from the collagen matrix, and both were extracted with 500 μL isopropanol and stored for 70 h refrigerated. The amount of extracted formazan was measured in duplicate for each sample by the OD_570_.

### Bovine Corneal Opacity and Permeability Test

The BCOP Assay was performed to evaluate the eye hazard potential of BondLynx, as measured by its ability to induce opacity and increase permeability in an isolated bovine cornea. The BCOP is an in vitro model that provides short-term maintenance of normal physiological and biological function of the bovine cornea in an isolated system so that eye damage can be assessed by quantitative measurements of changes in corneal opacity and permeability.^45,46^

Tests were performed using bovine corneas isolated from the eyes of young cattle after slaughter and transported in biological saline under cooled conditions. The positive control was 20% *w*/*v* imidazole in physiological saline and the negative control was physiological saline. BondLynx was ground with a mortar and pestle and applied directly to the corneas as a solid. Tests were performed in triplicate.

Bovine eyes were checked for unacceptable defects, such as opacity, scratches, pigmentation, and neovascularization upon receipt by removing them from the physiological saline and holding them in the light. Corneas were isolated from acceptable specimens and stored in a petri dish with cMEM (Eagle’s Minimum Essential Medium with 1% *v*/*v* L-glutamine and 1% *v*/*v* foetal bovine serum) at 32 ± 1°C. Isolated corneas were mounted in a corneal holder with the endothelial side against the O-ring of the posterior half of the holder and the anterior half of the holder tightened on top of the cornea with screws. The compartments of the corneal holder were filled with cMEM and the corneas were incubated for a minimum of 1 h at 32 ± 1°C. The initial corneal opacity was determined by replacing the medium in both compartments with fresh cMEM and using an opacitometer to measure the opacity against a cMEM filled chamber. Only corneas with an initial opacity <7 were used for testing.

A total of three samples were prepared for each test item by removing the medium from the anterior compartment of the holder and applying 750 μL of positive or negative control, or 306.6-333.6 mg of BondLynx. The corneal holders were positioned so that the test item uniformly covered the cornea within, and the entire assembly was incubated at 32 ± 1°C for 240 ± 10 min. The test item was then removed, and the epithelium was washed at least three times with MEM containing phenol red. Corneas were visually inspected for pH effects and dissimilar opacity patterns before refilling both compartments with fresh cMEM.

Corneal opacity was measured by the diminution of light (*I* = luminous flux per area) passing through the cornea. The opacity value was calculated according to the formula:

$$Opacity=\frac{\frac{I_{0}}{I}-0.9894}{0.0251}$$

where *I*_0_ is the empirically determined illuminance through a cornea holder with just windows and medium and *I* is the measured illuminance through a holder with a cornea. The change in opacity for each individual cornea was calculated by subtracting the initial opacity from the final post-treatment reading, and the corrected opacity was calculated by subtracting the average change in opacity of the negative control corneas (if > 0) from the change in opacity for corneas treated with BondLynx or positive control.

Following the final opacity measurement, permeability of the cornea to Na-fluorescein was evaluated by removing the medium from both corneal holder compartments, then refilling the posterior compartment with fresh cMEM and the anterior compartment with 1 mL of Na-fluorescein in cMEM (5 mg/mL). The corneal holders were positioned so that the test item uniformly covered the cornea within, and the entire assembly was incubated at 32 ± 1°C for 90 ± 5 min. The medium from the posterior compartment was then removed, and a 360 μL aliquot was placed in a 96-well plate to measure the OD_490_ in triplicate. Any sample with an OD_490_ that was 1.500 or higher was diluted to bring the OD_490_ into the acceptable range. The corrected OD_490_ was calculated by subtracting the average change in OD_490_ for the negative control (if > 0) from the change in OD_490_ of either BondLynx or positive control treated cornea.

The mean opacity and permeability values for each treatment group were used to calculate the in vitro irritancy score (IVIS) according to the formula below.

$$IVIS=Mean\;Opacity+15\left(Mean{OD}_{490}\right)$$

## Results and Discussion

### Bacterial Reverse Mutation Test

The mutagenic potential of BondLynx was assessed by the bacterial reverse mutation test (Ames test) using histidine-requiring strains of Salmonella typhimurium TA98 and TA100 in the presence and absence of an exogenous mammalian metabolic activation system (S9). BondLynx was tested with preincubation at a concentration range of 1.7 to 5000 μg/plate with dimethyl sulfoxide as the vehicle control and strain-specific positive controls.

In this study, acceptable responses were obtained for both controls by comparison to the historical control data range (Supplementary Table S1), indicating that the test conditions were adequate, and that the metabolic activation system functioned properly.

**Figure 2.**
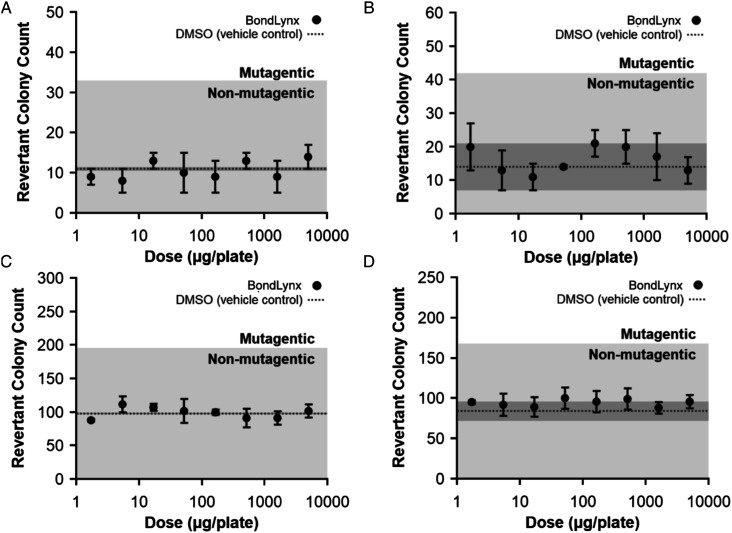
Mutagenic response of BondLynx in the *Salmonella typhimurium* reverse mutation assay in tester strand TA98 in the absence (A) or presence (B) of S9 metabolic mix, and in tester strand TA100 in the absence (C) or presence (D) of S9 metabolic mix. Data for the vehicle control (DMSO; mean of three plate counts) are presented as a dashed line, with shading in dark grey indicating standard deviation. The revertant count criteria for mutagenic activity are indicated with light grey shading.

BondLynx did not induce a dose-related increase in the number of revertant colonies in either of the two tester strains, in both the absence and presence of S9-metabolic activation (Figure 2 and Tables 1 and 2). As the total number of revertants for BondLynx was less than two times those observed in the concurrent vehicle control for strain TA100 and less than three times the concurrent vehicle control for strain TA98, BondLynx meets the criteria to be considered non-mutagenic. During testing, noticeable precipitation occurred at the highest concentration of BondLynx (5000 μg/plate) in the absence of S9 but did not influence automated counting of the plate. Additionally, a slight thinning of the microcolony lawn, indicating cytotoxicity, was observed at higher BondLynx concentrations in the absence of S9, but toxicity was not observed in the presence of S9 at any of the tested dose levels. Based on the results of this test, BondLynx is not mutagenic in the Salmonella typhimurium reverse mutation assay.

**Table 1.** Mutagenic Response of BondLynx in the *Salmonella typhimurium* Reverse Mutation Assay in Tester Strain TA98 in the Absence and Presence of S9 Metabolic Mix.^a,b^

Strain TA98		Revertant Colonies (Without S9)				Revertant Colonies (With S9)			
Compound	Dose (μg/plate)	A	B	C	Mean ± SD	A	B	C	Mean ± SD
BondLynx	1.7	8	11	7	**9 ± 2**	10	23	26	**20 ± 9**
	5.4	4	12	8	**8 ± 4**	4	15	19	**13 ± 8**
	17	15	11	12	**13 ± 4**	11	6	16	**11 ± 5**
	52	5	16	8	**10 ± 6**	15	14	14	**14 ± 1**
	164	8	14	5	**9 ± 5**	16	23	24	**21 ± 4**
	512	10	12	16	**13 ± 3**	15	19	26	**20 ± 6**
	1600	7	15	5	**9 ± 5**	26	16	10	**17 ± 8**
	5000	*18*	*12*	*12*	**14 ± 3**	8	16	16	**13 ± 5**
Vehicle control (DMSO)		10	11	11	**11 ± 1**	23	7	11	**14 ± 8**
2-nitrofluorene (+ control)	10	1962							
2-aminoanthracene (+ control)	1					657			

SD, standard deviation; DMSO, dimethyl sulfoxide; + control, positive control.

^a^Entries shaded in grey indicate slight thinning of the microcolony lawn.

^b^Italicized entries indicate that precipitation of BondLynx was observed but did not interfere with automated revertant counting.

**Table 2.** Mutagenic Response of BondLynx in the *Salmonella typhimurium* Reverse Mutation Assay in Tester Strain TA100 in the Absence and Presence of S9 Metabolic Mix.^a,b^

Strain TA100		Revertant Colonies (Without S9)				Revertant Colonies (With S9)			
Compound	Dose (μg/plate)	A	B	C	Mean ± SD	A	B	C	Mean ± SD
BondLynx	1.7	91	86	86	**88 ± 3**	93	94	**98**	**95 ± 3**
	5.4	127	110	98	**112 ± 15**	98	73	**105**	**92 ± 17**
	17	114	101	105	**107 ± 7**	72	98	**98**	**89 ± 15**
	52	105	122	78	**102 ± 22**	118	93	**88**	**100 ± 16**
	164	95	103	102	**100 ± 4**	83	113	**91**	**96 ± 16**
	512	84	110	79	**91 ± 17**	98	84	**116**	**99 ± 16**
	1600	82	86	105	**91 ± 12**	84	82	**97**	**88 ± 8**
	5000	*91*	*116*	*99*	**102 ± 13**	107	94	**88**	**96 ± 10**
Vehicle control (DMSO)		99	99	97	**98 ± 1**	86	69	**98**	**84 ± 15**
Methyl mesylate (+ control)	650	646							
2-aminoanthracene (+control)	5					363			

SD, standard deviation; DMSO, dimethyl sulfoxide; + control, positive control.

^a^Entries shaded in grey indicate slight thinning of the microcolony lawn.

^b^Italicized entries indicate that precipitation of BondLynx was observed but did not interfere with automated revertant counting.

### Cytotoxicity and Phototoxicity in 3T3 Fibroblasts Using the Neutral Red Uptake Assay

**Figure 3.**
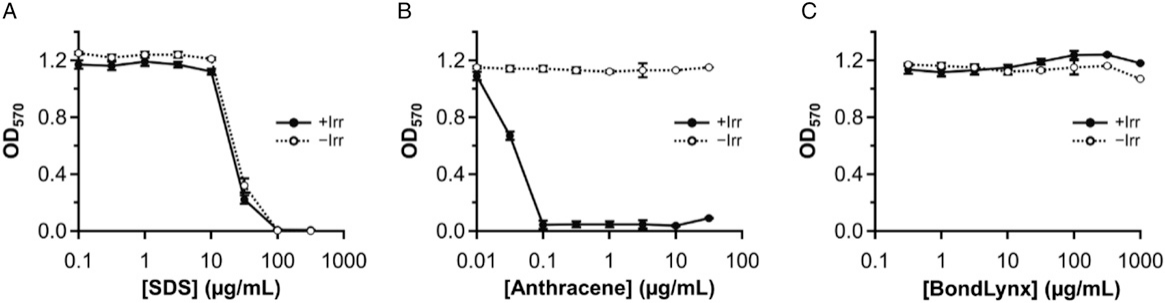
Neutral Red reuptake of SDS (A), anthracene (B), and BondLynx (C) in the presence (+Irr) and absence (–Irr) of UV-A irradiation. OD_570_ values are corrected with the appropriate vehicle control blank, and error bars represent the standard error of the mean.

BondLynx was evaluated for cytotoxicity and phototoxicity in the in vitro 3T3 NRU assay at concentrations ranging from .316 to 1000 μg/mL in ethanol, with SDS as the negative control and anthracene as the positive control. Where possible, the test item concentration causing 50% inhibition of Neutral Red uptake (IC_50_) was calculated from the plot of corrected OD_540_ vs concentration (Figure 3) with and without UV-A irradiation to determine the PIF.

In this study, the mean corrected OD_540_ values, for irradiated and non-irradiated vehicle controls were 1.16 and 1.13, respectively, resulting in a cell viability of 97%, falling above the acceptance criteria of 80%. The negative control IC_50_ values were 27.4 μg/mL and 21.65 μg/mL in the absence and presence of irradiation, respectively, resulting in a PIF of 1.14. The positive control showed an IC_50_ of .03 μg/mL in the presence of irradiation and no IC_50_ in the absence of irradiation, resulting in a PIF of >31.6. The IC_50_ and PIF values for both controls are comparable to historical data (Supplementary Table S2). It was therefore concluded that the test conditions were adequate, and that the test system functioned properly.

Spectroscopic measurements for the Neutral Red uptake assay (Supplementary Tables S3–S8) revealed no cytotoxicity after treatment of 3T3 fibroblasts with BondLynx over the range of concentrations tested. In both the presence and absence of UV-A irradiation, viability of cells exposed to all tested concentration of BondLynx approached or exceeded one hundred percent (99 ± 3% for Irr− and 105 ± 4% for Irr+). Since no change in viability occurred over the tested dosage range, no IC_50_ and PIF values could be calculated, leading to the classification of the test compound as non-phototoxic.

### Skin Corrosion Test Using a Human Skin Model

The ability of pure BondLynx to induce skin corrosion on a human three-dimensional epidermal model was evaluated after 3 min and 1 h exposure times. Cytotoxicity was assessed from the reduction of mitochondrial dehydrogenase activity, as indicated by formazan production (measured spectroscopically as OD_570_) from MTT. BondLynx was applied directly to the skin model, with Milli-Q water serving as the negative control and 8.0 N aqueous potassium hydroxide (KOH) solution as the positive control.

Addition of BondLynx to Milli-Q water and isopropanol resulted in corrected OD_570_ values of .0041 and − .0011, respectively, indicating that the presence of BondLynx did not interfere with the spectroscopic detection of formazan. Additionally, no colour change was observed when mixing BondLynx with MTT, indicating that the BondLynx did not directly reduce MTT and interfere with the MTT endpoint. The absolute mean OD_570_ of the negative control tissues (treated with Milli-Q water) was within the acceptance limits of OECD 431 (.8-2.8) and the laboratory historical control data range (Supplementary Table S9). In this study, the mean relative tissue viability following 1 h exposure to the positive control was 5.9%. In the range of 20%-100% cell viability, the coefficient of variation between tissue replicates was ≤12%, indicating that the test system functioned properly.

Skin corrosion is expressed as the remaining cell viability after exposure to the test items (Table 3). Viabilities of 101% and 95% were obtained following 3 min and 1 h exposure to BondLynx, respectively. The mean relative tissue viability of >50% following 3 min exposure, and >15% following 1 h exposure indicated that BondLynx is not corrosive.

**Table 3.** Absorption and Cell Viability Following Exposure to BondLynx in the In Vitro Skin Corrosion Test.

		3 min Exposure				1 h Exposure			
Material		OD_570_	OD_570_ corrected	Mean ± SD	Viability (%)	OD_570_	OD_570_ corrected	Mean ± SD	Viability (%)
Milli-Q (− control)	A	1.6727	1.6278	1.7 ± 0.2	**100**	2.1106	2.0657	1.9 ± 0.1	**100**
		1.6519	1.607			2.0617	2.0168		
		1.6602	1.6153			2.0108	1.9659		
	B	1.8795	1.8346			1.9656	1.9207		
		1.8697	1.8248			1.9109	1.866		
		1.8758	1.8309			1.8918	1.8469		
8.0 N KOH (+ control)	A	.1679	.123	.11 ± .01	**6.5**	.1607	.1158	.114 ± .001	**5.9**
		.1674	.1225			.1577	.1128		
		.1673	.1224			.1578	.1129		
	B	.1478	.1029			.1595	.1146		
		.1479	.103			.1604	.1155		
		.1469	.102			.1591	.1142		
BondLynx	A	1.8819	1.837	1.7 ± 0.1	**101**	1.9919	1.947	1.85 ± .09	**95**
		1.869	1.8241			1.9396	1.8947		
		1.8445	1.7996			1.9528	1.9079		
	B	1.7365	1.6916			1.8727	1.8278		
		1.7069	1.662			1.8128	1.7679		
		1.7026	1.6577			1.8033	1.7584		

SD, standard deviation; − control, negative control; + control, positive control; KOH, potassium hydroxide.

### Skin Irritation Test Using a Human Skin Model

The ability of pure BondLynx to induce skin irritation on a human three-dimensional epidermal model was evaluated following 15 min exposure. Cytotoxicity was assessed 42 h after exposure from the reduction of mitochondrial dehydrogenase activity, as indicated by formazan production (measured spectroscopically as OD_570_) from MTT.

BondLynx did not cause a colour change or precipitation in the presence of MTT, and exhibited an OD_570_ ≤ .08 in solution, indicating that it did not interfere with the MTT endpoint or spectroscopic detection of formazan. The positive control, 5% SDS, had a mean cell viability of 4% following exposure, and the absolute mean OD_570_ of the negative control, phosphate buffered saline, was within the laboratory historical control range (Supplementary Table S10). The standard deviation value of the percentage viability of three tissues treated identically was <3%, indicating that the test system functioned properly.

Skin irritation is expressed as the remaining cell viability following a 15 min exposure to the test item, and a 42-h incubation after the test item was washed off (Table 4). The relative mean cell viability in the epidermal model following exposure to BondLynx was 106%. Since viability was >50%, BondLynx is considered to be a non-irritant.

**Table 4.** Absorption and Cell Viability Following Exposure to BondLynx in the In Vitro Skin Irritation Test.

Material	Replicate	OD_570_	OD_570_ Corrected	Mean ± SD	Cell Viability (%)
Phosphate buffered saline (− control)	A	1.0096	.9667	.963 ± .022	**100**
		.9994	.9565		
	B	.9910	.9481		
		.9788	.9359		
	C	1.0350	.9921		
		1.0237	.9808		
5% SDS (+control)	A	.0783	.0354	.039 ± .005	**4.0**
		.0796	.0367		
	B	.0868	.0439		
		.0878	.0449		
	C	.0796	.0367		
		.0776	.0347		
BondLynx	A	1.0621	1.0192	1.019 ± .011	**106**
		1.0517	1.0088		
	B	1.0668	1.0239		
		1.0420	.9991		
	C	1.0901	1.0472		
		1.0602	1.0173		

SD, standard deviation; − control, negative control; + control, positive control; SDS, sodium dodecyl sulphate.

### Bovine Corneal Opacity and Permeability Test

The eye hazard potential of BondLynx was measured by its ability to induce opacity and increase permeability in isolated bovine corneas. Damage to the corneas was assessed by the quantitative measurement of changes in corneal opacity, and by the spectroscopic detection of sodium fluorescein (OD_490_) which permeated the corneas over 90 min, following a 240 min exposure to the test items. The IVIS was calculated according to OECD guidelines to determine the UN GHS eye damage classification.

The individual IVIS for the negative control, physiological saline, ranged from −1.9 to −.5, less than the upper limits of the historical mean (Supplementary Table S11), and the corneas were clear after 240 min of treatment. The individual IVIS for the positive control, 20% imidazole, ranged from 133 to 143, within two standard deviations of the historical mean, and the corneas were turbid after treatment. It was therefore concluded that the test conditions were adequate, and that the test system functioned properly.

The corneas treated with BondLynx were clear after treatment, with opacity values ranging from .7 to 1.5 (Supplementary Table S12), and permeability values ranging from .000 to .019 (Supplementary Table S13). No pH effects associated with BondLynx were observed in the rinsing medium, and IVIS scores ranging from .9 to 1.5 were obtained (Table 5). The IVIS cut-off for identifying test items inducing serious eye damage (UN GHS Category 1) is > 55, while scores 3-55 do not allow for prediction of eye damage, and scores <3 indicate that no classification for eye irritation or damage is required (UN GHS No Category). With a IVIS score of 1.2, BondLynx does not require classification for eye damage or irritation under UN GHS standards.

**Table 5.** Summary of Opacity, Permeability, and IVIS Scores for the Bovine Corneal Opacity and Permeability Test of BondLynx.

Material	Opacity	OD_490_	IVIS	Mean IVIS ± SD
Physiological saline (− control)	−1.9	.003	−1.9	**−1.2 ± 0.7**
	−0.9	.021	−0.5	
	−1.3	.006	−1.2	
20% imidazole (+control)	115	1.861	143	**137 ± 5**
	108	1.613	133	
	100	2.413	136	
BondLynx	1.5	.000	1.5	**1.2 ± 0.4**
	0.9	.002	0.9	
	0.7	.019	1.0	

SD, standard deviation; − control, negative control; + control, positive control; IVIS, in vitro irritancy score.

## Conclusion

The evaluation of BondLynx toxicity was carried out by the bacterial reverse mutation assay in tester strains TA98 and TA100 (to measure mutagenesis potential), the Neutral Red uptake assay on 3T3 fibroblasts (to measure phototoxicity), skin corrosion and skin irritation tests on a human skin model, and BCOP tests (to measure eye damage and irritation). The bacterial reverse mutation assay revealed no increase in revertant colonies with increasing dosage, and revertant colony counts well below cut-off values at all doses tested, indicating that BondLynx is non-mutagenic. In the Neutral Red uptake assay, no PIF could be calculated, as no cytotoxicity was observed at any concentration tested (mean cell viability 97%), classifying BondLynx as non-phototoxic. The relative mean tissue viability compared to the negative control (Milli-Q water) for the human skin model exposed to BondLynx was 101% after 3 min exposure and 95% after 1 h exposure, indicating that BondLynx is non-corrosive, and the viability of 106% relative to the negative control (phosphate buffered saline) 42 h after a 15 min exposure indicates that BondLynx is also non-irritant. Finally, with an IVIS of 1.2 from the bovine cornea opacity and permeability test, BondLynx does not cause serious eye damage or irritation.

Taken together these results recommend diazirine-based molecular adhesives over more common carbene precursors, such as diazoalkanes, with known cytotoxic effects.^47^ Existing studies of the leachates from diazirine grafted biomaterials following UV-activation additionally reveal no genotoxic or sensitization effects from the degradation products of diazirine-mediated crosslinking.^48^ Initial assessment of BondLynx toxicity suggest a desirable safety profile for the handling and application of diazirine-based molecular adhesives. Looking forward, additional safety concerns such a skin sensitization could be evaluated in vitro (eg OECD Test No. 442), or in vivo along with more stringent biocompatibility studies if medical device applications are explored in the future. In silico methods such as quantitative structure activity relationships (QSAR) could also be employed to predict acute, chronic, and organ-specific toxicity and support the findings presented, and in vivo studies could be employed to corroborate in vitro findings and provide accurate, physiologically relevant dose-response data.^49^

## Supplemental Material

Supplemental Material - Safety Evaluation of a Prototypical Diazirine-Based Covalent Crosslinker and Molecular Adhesive Supplemental Material for Safety Evaluation of a Prototypical Diazirine-Based Covalent Crosslinker and Molecular Adhesive by Miranda J. Baran, Rebecca Hof, Angelique Groot, Irene Eurlings, Jet Gijsbrechts, Britt de Jong, and Jeremy E. Wulff in International Journal of Toxicology
