# Supplementary material for: Safety Evaluation of a Prototypical Diazirine-Based Covalent Crosslinker and Molecular Adhesive
Source: Int J Toxicol. 2023 Nov 21;43(2):146–56. doi: 10.1177/10915818231215692 (PMC10916352; doi:10.1177/10915818231215692)
Supplement: Supplemental Material - Safety Evaluation of a Prototypical Diazirine-Based Covalent Crosslinker and Molecular Adhesive [file sj-pdf-1-ijt-10.1177_10915818231215692.pdf]

Supplementary Materials

Materials and Instrumentation

2,2-bis[4-[3-(trifluoromethyl)-3*H*-diazirin-3-yl]phenyl]hexafluoropropane (BondLynx) was prepared according to literature procedures,[9] and purity was confirmed by ^{1}H-NMR, ^{19}F-NMR, and UPLC (Figures S1–S3, respectively). TA98 and TA100 tester strains, and S9-mix, were acquired from Trinova Biochem GmbH. Dimethyl sulfoxide (DMSO), top agar, ethanol, potassium hydroxide (KOH), phosphate buffer saline (PBS) and isopropanol for skin irritation studies, physiological saline, and imidazole were acquired from Merck KGaA. 2-nitrofluorene, methyl mesylate, 2-aminoanthracene, sodium dodecyl sulfate (SDS, >99.0% and 5% aq solution), Neutral Red, 3-(4,5-dimethylthiazol-2-yl)-2,5-diphenyl tetrazolium bromide (MTT), Na-fluorescein, and trifluoroacetic acid (HPLC grade) were acquired from Sigma-Aldrich. Phosphate buffer for mutagenesis studies was acquired from Euronet Animal Health. Anthracene (99.9%) was acquired from Fluka Chemie. Balb/c 3T3 firbroblasts (clone 31) were acquired from American Type Culture Collection. Earle's Balanced Salt Solution (EBSS) medium, Dulbeco’s Modified Eagle Medium (DMEM) for phototoxicity studies, Eagle’s Minimum Essential Medium (cMEM), foetal calf serum, L-glutamine, penicillin, streptomycin, Dulbecco's phosphate-buffered saline (DPBS) and fetal bovine serum were acquired from Life Technologies. Milli-Q water was acquired from Millipore Corporation. Phosphate buffer saline (PBS) for skin corrosion studies was acquired from Invitrogen Corporation. Supplemented Dulbeco’s Modified Eagle Medium (DMEM), isopropanol, and EpiDerm Skin Model (EPI-200, Lot 33079, kit O) for skin corrosion studies were acquired from MatTek Corporation. EPISKIN Small Model (Batch 20 EKIN 036), maintenance medium, and assay medium were acquired from SkinEthic Laboratories. Bovine eyes were acquired from Vitelco. Corneal holders were acquired from Duratec Analysentechnik GmbH. Acetonitrile (HPLC grade) was acquired from Fisher Scientific.

NMR spectra (^{1}H and ^{19}F) were recorded on a Bruker Avance-III 300 MHz NMR spectrometer and processed using Bruker TopSpin 4.0.1 Software. Ultra-performance liquid chromatography was acquired using a Shimadzu Nexera X2 instrument with a Waters ACQUITY UPLC® BEH C18 column (2.1 × 30 mm, particle size 1.7 µm). Irradiation of “Irr+” cultures for the phototoxicity study was performed with the Oriel's Sol UV™ solar simulator from Newport Corporation. All spectroscopic measurements were performed with an Infinite® M200 Pro Plate Reader from TECAN Trading AG. Corneal opacities were measured using the BASF-OP3.0 opacitometer from Duratec Analysentechnik GmbH.

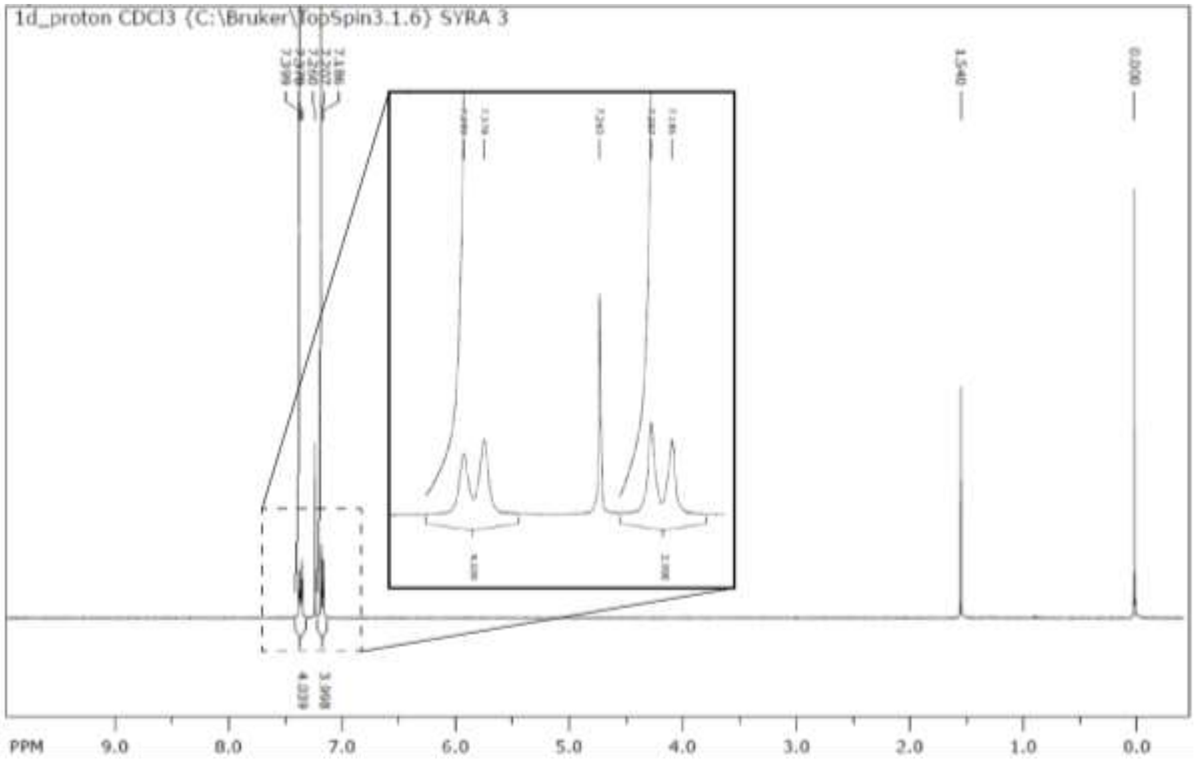

Figure S1. ^{1}H-NMR of 2,2-bis[4-[3-(trifluoromethyl)-3*H*-diazirin-3-yl]phenyl]hexafluoropropane (BondLynx) acquired in $CDCl_3$ on a 400 MHz spectrometer. The inlay shows an expansion of the aromatic signals.

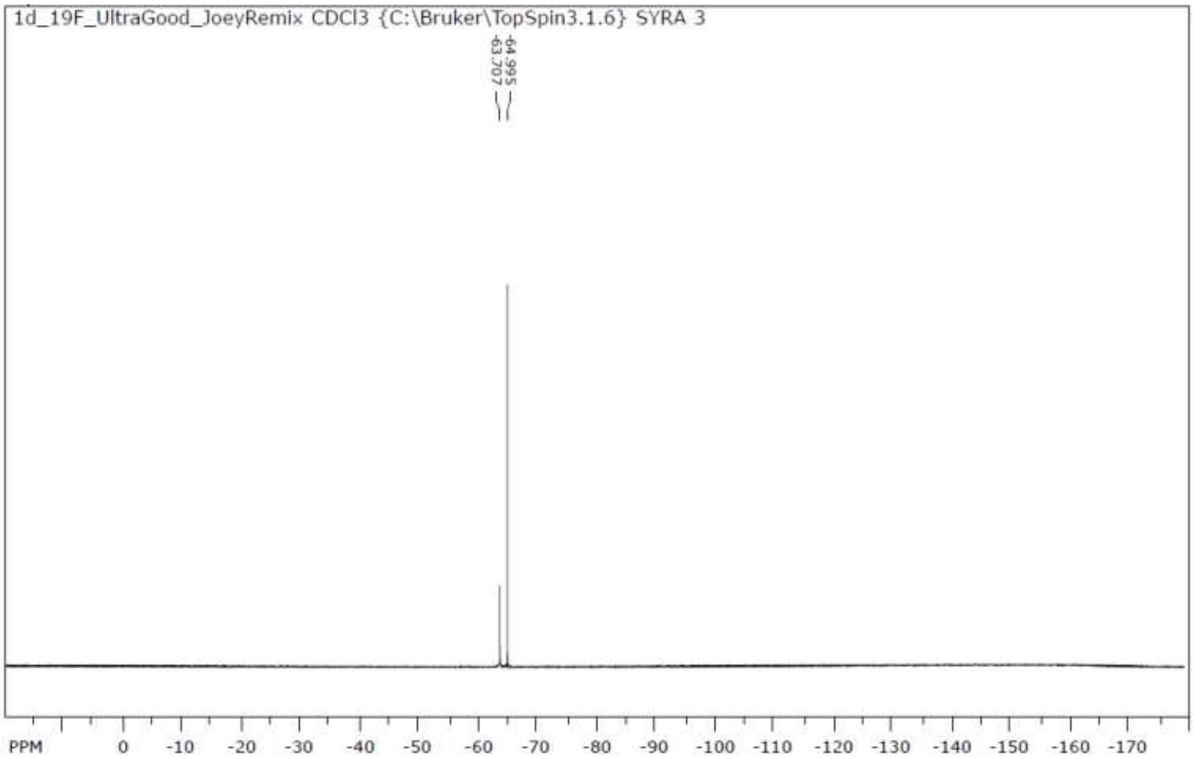

Figure S2. ^{19}F-NMR of 2,2-bis[4-[3-(trifluoromethyl)-3*H*-diazirin-3-yl]phenyl]hexafluoropropane (BondLynx) acquired in $CDCl_3$ on a 400 MHz spectrometer.

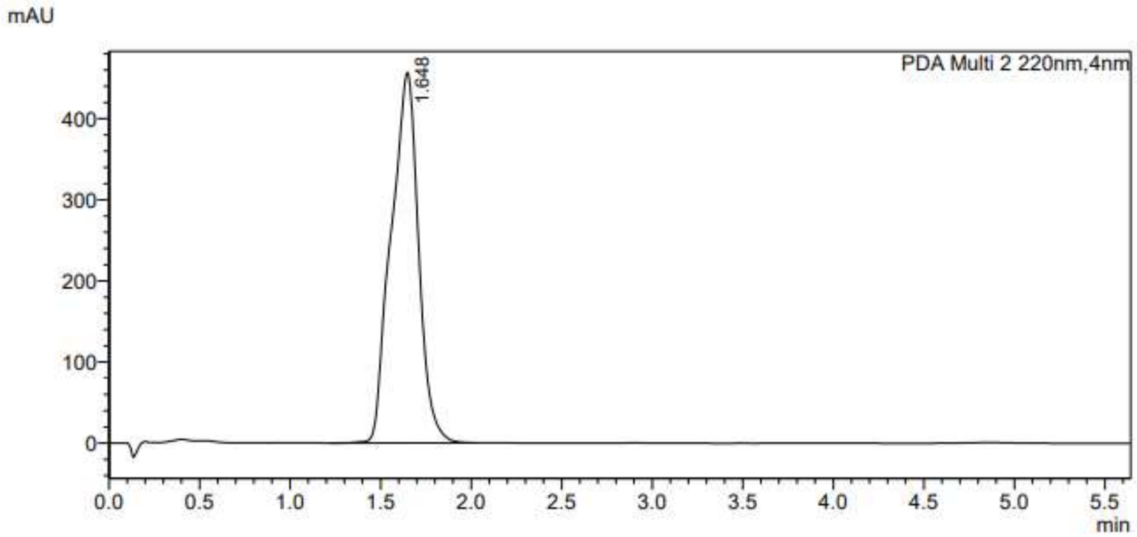

Figure S3. UPLC trace of BondLynx. Waters ACQUITY UPLC® BEH C18 column (2.1 × 30 mm, particle size 1.7 µm) at 40 °C oven temperature and a detection wavelength of 220 nm. Eluent A: MilliQ water with 0.05% trifluoroacetic acid (TFA); eluent B: acetonitrile with 0.05% trifluoroacetic acid (TFA); isocratic (25:75) over 6 min with a flow rate of 0.7 mL·min^{-1}; injection volume: 10 µL; retention time: 1.65 min; peak area: 99.86%. The data was processed using LabSolutions.

Historical Data and Individual Spectroscopic Measurements

Table S1. Historical data (June 2017 – June 2020) for the bacterial reverse mutation assay.

	Positive Control				Solvent Control			
	TA98		TA100		TA98		TA100	
	+S9	–S9	+S9	–S9	+S9	–S9	+S9	–S9
Revertant Count Range	258 – 2033	365 – 2118	397 – 2666	439 – 1993	8 – 60	5 – 61	55 – 176	61 – 188
Revertant Count Mean ± SD	903 ± 367	1220 ± 221	1371 ± 412	879 ± 170	19 ± 6	14 ± 5	103 ± 21	110 ± 18
n	2621	2670	2634	2654	2754	2717	2724	2806

Abbreviations: +S9, with S9 mix; –S9, without S9 mix; SD, standard deviation; n, number of observations.

Table S2. Historical data (Jan 2016 – Jun 2020) for the Neutral Red uptake assay of 3T3 fibroblasts cells.

	Positive Control			Negative Control		
	IC_{50} +Irr (µg/mL)	IC_{50} –Irr (µg/mL)	PIF	IC_{50} +Irr (µg/mL)	IC_{50} –Irr (µg/mL)	PIF
Range	NA	0.01 – 0.16	201 – 3162	11.4 – 38.8	11.0 – 37.4	0.66 – 1.52
Mean ± SD	31.62 ± 0.0	0.04 ± 0.03	1229 ± 731	23.6 ± 7.6	21.6 ± 7.0	1.11 ± 0.20
n	35	35	35	35	35	35

Abbreviations: IC_{50}, concentration of test chemical causing 50% inhibition of Neutral Red uptake; +Irr, with irradiation; –Irr, without irradiation; SD, standard deviation; n, number of observations.

Table S3. Individual OD_{540} values after exposure to sodium dodecyl sulfate (negative control) with UV-A irradiation.

	Concentration (µg/mL)								Vehicle Control		Blank	
	316	100	31.6	10	3.16	1	0.316	0.1				
OD_{540}	0.0519	0.0628	0.1728	1.1033	1.1014	1.1578	1.1307	1.0577	1.1999	1.2305	0.0503	0.0491
	0.0620	0.0585	0.1771	1.0759	1.1423	1.1367	1.1287	1.1698	1.1898	1.0759	0.0677	0.0489
	0.0572	0.0524	0.2642	1.1899	1.2474	1.1530	1.1355	1.1896	1.1605	1.1995	0.0516	0.0531
	0.0615	0.0528	0.1926	1.1900	1.1883	1.2109	1.1959	1.1587	1.2278	1.2651	0.0528	0.0557
	0.0524	0.0524	0.2878	1.2023	1.2633	1.2727	1.2836	1.2960	1.2565	1.1595	0.0509	0.0504
	0.0642	0.0521	0.2476	1.2415	1.2643	1.3513	1.2427	1.2947	1.2892	1.2901	0.0512	0.0496
	0.0516	0.0834	0.4113	1.2636	1.2826	1.3687	1.3227	1.3175	1.1788	1.2201	0.0505	0.0517
	0.0601	0.0524	0.4442	1.0800	1.2919	1.2651	1.2620	1.2701	1.2554	1.3257	0.0649	0.0491
Mean	0.0576	0.0584	0.2747	1.1683	1.2227	1.2395	1.2127	1.2193	1.2203		0.0530	
**Corrected Mean**	**0.006**	**0.007**	**0.22**	**1.12**	**1.17**	**1.19**	**1.16**	**1.17**	**1.17**			
Viability (%)	0.5	0.6	18.8	95.7	100.0	101.7	99.1	100.0				
SEM	0.002	0.004	0.03	0.02	0.02	0.03	0.03	0.03	0.01			

Abbreviations: SEM, standard error of the mean.

Table S4. Individual OD_{540} values after exposure to sodium dodecyl sulfate (negative control) without UV-A irradiation.

	Concentration (µg/mL)								Vehicle Control		Blank	
	316	100	31.6	10	3.16	1	0.316	0.1				
OD_{540}	0.0521	0.0520	0.3430	1.2271	1.2967	1.2253	1.1981	1.2973	1.2466	1.2631	0.0496	0.0517
	0.0512	0.0532	0.2752	1.2507	1.2375	1.1941	1.2659	1.2635	1.2518	1.2457	0.0493	0.0525
	0.0524	0.0519	0.2318	1.2511	1.2175	1.2933	1.2247	1.2392	1.2482	1.2676	0.0518	0.0510
	0.0516	0.0516	0.2469	1.3004	1.2685	1.2901	1.2569	1.2740	1.2419	1.2884	0.0511	0.0513
	0.0509	0.0513	0.2299	1.3130	1.3223	1.2827	1.2901	1.3487	1.3326	1.2999	0.0505	0.0499
	0.0514	0.0660	0.5418	1.2871	1.3474	1.3739	1.3300	1.3456	1.3056	1.2648	0.0556	0.0501
	0.0526	0.0541	0.5879	1.2051	1.2831	1.3360	1.3679	1.3422	1.2901	1.2752	0.0507	0.0522
	0.0529	0.0527	0.5237	1.2691	1.3353	1.3132	1.2662	1.3089	1.3279	1.2768	0.0564	0.0486
Mean	0.0519	0.0541	0.3725	1.2630	1.2885	1.2886	1.2750	1.3024	1.2766		0.0514	
**Corrected Mean**	**0.0005**	**0.003**	**0.32**	**1.21**	**1.24**	**1.24**	**1.22**	**1.25**	**1.225**			
Viability (%)	0.04	0.2	26.1	98.8	101.2	101.2	99.6	102.0				
SEM	0.0002	0.002	0.05	0.01	0.02	0.02	0.02	0.01	0.007			

Abbreviations: SEM, standard error of the mean.

Table S5. Individual OD_{540} values after exposure to anthracene (positive control) with UV-A irradiation.

	Concentration (µg/mL)								Vehicle Control		Blank	
	31.6	10	3.16	1	0.316	0.1	0.0316	0.01				
OD_{540}	0.1319	0.0809	0.0820	0.0776	0.0926	0.0775	0.6670	1.0532	1.1100	1.1295	0.0506	0.0507
	0.1353	0.0993	0.1196	0.1120	0.0970	0.0735	0.7347	1.1361	1.1480	1.1465	0.0518	0.0501
	0.1383	0.0876	0.0875	0.1051	0.0933	0.1179	0.7336	1.1619	1.1597	1.1531	0.0613	0.0564
	0.1442	0.0884	0.1088	0.0869	0.0997	0.0901	0.6394	1.1106	1.1463	1.1519	0.0609	0.0517
	0.1440	0.0849	0.1098	0.1179	0.0894	0.0793	0.6526	1.1585	1.1772	1.2038	0.0537	0.0517
	0.1382	0.0854	0.1035	0.1179	0.1464	0.0796	0.6997	1.1679	1.1670	1.1704	0.0526	0.0576
	0.1613	0.0874	0.0946	0.0917	0.0856	0.1445	0.7894	1.1931	1.2176	1.1789	0.0522	0.0519
	0.1293	0.0896	0.0784	0.0767	0.0777	0.0926	0.8806	1.1528	1.2160	1.1467	0.0506	0.0498
Mean	0.1403	0.0879	0.0980	0.0982	0.0977	0.0944	0.7246	1.1418	1.1639		0.0534	
**Corrected Mean**	**0.089**	**0.037**	**0.047**	**0.047**	**0.046**	**0.043**	**0.67**	**1.09**	**1.111**			
Viability (%)	8.01	3.3	4.2	4.2	4.1	3.9	60.3	98.1				
SEM	0.003	0.002	0.005	0.006	0.007	0.008	0.03	0.01	0.007			

Abbreviations: SEM, standard error of the mean.

Table S6. Individual OD_{540} values after exposure to anthracene (positive control) without UV-A irradiation.

	Concentration (µg/mL)								Vehicle Control		Blank	
	31.6	10	3.16	1	0.316	0.1	0.0316	0.01				
OD_{540}	1.2290	1.1195	1.0891	1.0977	1.0303	1.0334	1.0401	1.0860	1.1305	1.0917	0.0498	0.0481
	1.1678	1.2226	1.1691	1.1649	1.1776	1.2063	1.2252	1.1565	1.1381	1.1434	0.0485	0.0493
	1.1577	1.2494	1.2011	1.1537	1.1831	1.1961	1.2048	1.2484	1.1961	1.1514	0.0503	0.0490
	1.1553	1.2426	1.1936	1.1795	1.1877	1.1754	1.2133	1.2004	1.1871	1.1434	0.0492	0.0478
	1.1777	1.1296	1.0773	1.2066	1.2246	1.1972	1.1646	1.1864	1.1836	1.1595	0.0499	0.0486
	1.2652	1.1552	1.2198	1.1926	1.1563	1.2524	1.2704	1.2222	1.2037	1.1834	0.0537	0.0487
	1.1830	1.1763	1.2679	1.1962	1.2691	1.2586	1.2651	1.2600	1.2230	1.1759	0.0494	0.0508
	1.2507	1.1664	1.2360	1.1732	1.2256	1.1784	1.1636	1.2260	1.1988	1.1838	0.0506	0.0476
Mean	1.1983	1.1827	1.1817	1.1706	1.1818	1.1872	1.1934	1.1982	1.1683		0.0495	
**Corrected Mean**	**1.15**	**1.13**	**1.13**	**1.12**	**1.13**	**1.14**	**1.14**	**1.15**	**1.119**			
Viability (%)	102.77	101.0	101.0	100.1	101.0	101.9	101.9	102.8				
SEM	0.01	0.02	0.02	0.01	0.02	0.02	0.02	0.02	0.008			

Abbreviations: SEM, standard error of the mean.

Table S7. Individual OD_{540} values after exposure to BondLynx with UV-A irradiation.

	Concentration (µg/mL)								Vehicle Control		Blank	
	1000	316	100	31.6	10	3.16	1	0.316				
OD_{540}	1.1658	1.2230	1.2729	1.1944	1.1886	1.1638	1.1231	1.1671	1.1218	1.1396	0.0482	0.0479
	1.2321	1.1363	1.2716	1.2218	1.2115	1.2002	1.1680	1.1338	1.1128	1.1696	0.0475	0.0476
	1.2594	1.3671	1.2569	1.2404	1.2041	1.1592	1.1658	1.1830	1.0980	1.1794	0.0486	0.0486
	1.2656	1.3610	1.2821	1.2327	1.1877	1.1832	1.1677	1.1757	1.1958	1.1651	0.0487	0.0475
	1.2363	1.3391	1.3331	1.2533	1.1972	1.1565	1.2031	1.2163	1.1837	1.1740	0.0492	0.0490
	1.2370	1.3080	1.2978	1.2433	1.2137	1.2449	1.1852	1.2146	1.1645	1.2003	0.0502	0.0622
	1.2048	1.3152	1.3032	1.3179	1.1847	1.1651	1.1691	1.2038	1.2151	1.1813	0.0501	0.0494
	1.2205	1.2683	1.2839	1.2083	1.2174	1.1935	1.1538	1.1936	1.2922	1.1900	0.0483	0.0474
Mean	1.2277	1.2898	1.2877	1.2390	1.2006	1.1833	1.1670	1.1860	1.1740		0.0494	
**Corrected Mean**	**1.18**	**1.24**	**1.236**	**1.19**	**1.149**	**1.13**	**1.116**	**1.135**	**1.12**			
Viability (%)	105.36	110.7	110.4	106.3	102.6	100.9	99.6	101.3				
SEM	0.01	0.03	0.008	0.01	0.004	0.01	0.008	0.009	0.01			

Abbreviations: SEM, standard error of the mean.

Table S8. Individual OD_{540} values after exposure to BondLynx without UV-A irradiation.

	Concentration (µg/mL)								Vehicle Control		Blank	
	1000	316	100	31.6	10	3.16	1	0.316				
OD_{540}	0.9572	0.9715	1.1191	1.0711	1.0238	1.1185	1.1440	1.1590	1.1393	1.2231	0.0471	0.0618
	1.1715	1.1621	1.2035	1.1825	1.0663	1.1459	1.2513	1.1873	1.1830	1.1875	0.0575	0.0463
	1.0188	1.2150	1.1795	1.1791	1.1871	1.2308	1.2075	1.2126	1.1577	1.1717	0.0478	0.0468
	1.1429	1.3256	1.2269	1.2304	1.1624	1.2048	1.1732	1.2130	1.1948	1.1425	0.0480	0.0463
	1.1545	1.2809	1.2593	1.2279	1.2436	1.2476	1.2523	1.2394	1.2048	1.1462	0.0491	0.0516
	1.1541	1.2398	1.1960	1.2122	1.2261	1.2743	1.2510	1.2620	1.1964	1.1957	0.0480	0.0474
	1.1659	1.2591	1.2049	1.2050	1.2475	1.2371	1.2180	1.2836	1.2304	1.2416	0.0483	0.0475
	1.1990	1.2136	1.1881	1.1353	1.1966	1.1676	1.2003	1.2335	1.2810	1.2498	0.0474	0.0476
Mean	1.1205	1.2085	1.1972	1.1804	1.1692	1.2033	1.2122	1.2238	1.1966		0.0493	
**Corrected Mean**	**1.07**	**1.16**	**1.15**	**1.13**	**1.12**	**1.15**	**1.16**	**1.17**	**1.15**			
Viability (%)	93.04	100.9	100.0	98.3	97.4	100.0	100.9	101.7				
SEM	0.03	0.04	0.01	0.02	0.03	0.02	0.01	0.01	0.01			

Abbreviations: SEM, standard error of the mean.

Table S9. Historical data (Dec 2016 – June 2020) for the in vitro skin corrosion study.

	Negative Control		Positive Control	
	OD_{570} 3 min treatment	OD_{570} 1h treatment	OD_{570} 3 min treatment	OD_{570} 1h treatment
Range	1.258 – 2.414	1.317 – 2.361	0.080 – 0.671	0.032 – 0.319
Mean ± SD	1.737 ± 0.197	1.758 ± 0.185	0.175 ± 0.084	0.138 ± 0.046
n	152	152	150	150

Abbreviations: SD, standard deviation; n, number of observations.

Table S10. Historical data (June 2017 – June 2020) for the in vitro skin irritation study.

	Negative Control OD_{570}	Positive Control OD_{570}
Range	0.422 – 1.426	0.027 – 0.449
Mean ± SD	1.010 ± 0.179	0.105 ± 0.080
n	141	141

Abbreviations: SD, standard deviation; n, number of observations.

Table S11. Historical data (June 2017–June 2020) for the bovine corneal opacity and permeability study.

	Negative Control			Positive Control
	Opacity	Permeability (OD_{490})	IVIS	IVIS
Range	–2.60 – 6.20	–0.011 – 0.081	–2.70 – 6.30	86 – 251
Mean ± SD	1.18 ± 1.76	0.012 ± 0.013	1.37 ± 1.82	150 ± 28
n	170	170	170	174

Abbreviations: IVIS, in vitro irritancy score; SD, standard deviation; n, number of observations.

Table S12. Individual opacity data for the bovine corneal opacity study.

Material	Initial Opacity	Final Opacity	Opacity Change	Corrected Opacity Change	**Mean Opacity Change**
Physiological Saline (negative control)	3.2<br>2.6<br>2.4	1.3<br>1.8<br>1.1	-1.9<br>-0.8<br>-1.3		**-1.3**
20% Imidazole (positive control)	2.5<br>2.3<br>4.1	117.3<br>110.8<br>103.7	114.8<br>108.5<br>99.6	114.8<br>108.5<br>99.6	**108**
BondLynx	0.3<br>0<br>1.8	1.9<br>0.9<br>2.5	1.6<br>0.9<br>0.7	1.6<br>0.9<br>0.7	**1.1**

Table S13. Individual OD_{490} values for the bovine corneal permeability study.

Material	Replicate	Dilution	OD_{490}	Corrected OD_{490}	Mean OD_{490}	**Undiluted OD_{490}**
Physiological Saline (negative control)	A	1	0.001<br>0.005<br>0.004		0.003	**0.003**
	B	1	0.020<br>0.036<br>0.007		0.021	**0.021**
	C	1	-0.002<br>0.011<br>0.010		0.006	**0.006**
20% Imidazole (positive control)	A	6	0.326<br>0.318<br>0.317	0.316<br>0.308<br>0.307	0.310	**1.861**
	B	6	0.282<br>0.278<br>0.277	0.272<br>0.268<br>0.267	0.269	**1.613**
	C	6	0.417<br>0.411<br>0.409	0.407<br>0.401<br>0.399	0.402	**2.413**
BondLynx	A	1	0.017<br>0.007<br>0.006	0.007<br>-0.003<br>-0.004	0.000	**0.000**
	B	1	0.011<br>0.014<br>0.011	0.001<br>0.004<br>0.001	0.002	**0.002**
	C	1	0.042<br>0.022<br>0.023	0.032<br>0.012<br>0.013	0.019	**0.019**
